# Impact of Surface Functionalization and Deposition Method on Cu-BDC surMOF Formation, Morphology, Crystallinity, and Stability

**DOI:** 10.1021/acs.langmuir.3c01505

**Published:** 2023-08-16

**Authors:** B. Dulani Dhanapala, Dayton L. Maglich, Mary E. Anderson

**Affiliations:** Department of Chemistry, Furman University, Greenville, South Carolina 29613, United States

## Abstract

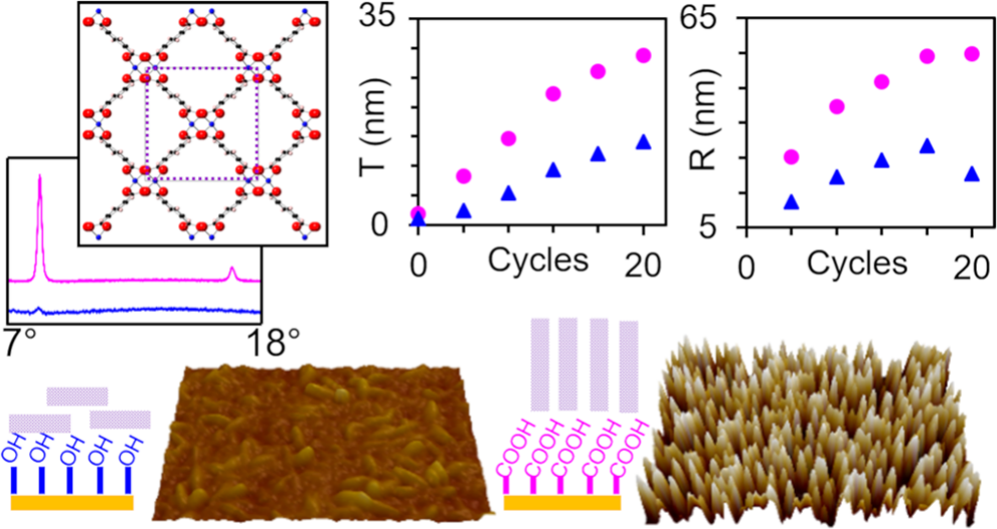

For direct integration into device architectures, surface–anchored metal–organic framework (surMOF) thin films are attractive systems for a wide variety of electronic, photonic, sensing, and gas storage applications. This research systematically investigates the effect of deposition method and surface functionalization on the film formation of a copper paddle-wheel-based surMOF. Solution-phase layer-by-layer (LBL) immersion and LBL spray deposition methods are employed to deposit copper benzene-1,4-dicarboxylate (Cu-BDC) on gold substrates functionalized with carboxyl- and hydroxyl-terminated alkanethiol self-assembled monolayers (SAMs). A difference in crystal orientation is observed by atomic force microscopy and X-ray diffractometry based on surface functionalization for films deposited by the LBL immersion method but not for spray-deposited films. Cu-BDC crystallites with a strong preferred orientation perpendicular to the substrate were observed for the films deposited by the LBL immersion method on carboxyl-terminated SAMs. These crystals could be removed upon testing adhesive properties, whereas all other Cu-BDC surMOF film structures demonstrated excellent adhesive properties. Additionally, film stability upon exposure to water or heat was investigated. Ellipsometric data provide insight into film formation elucidating 7 and 14 Å average thicknesses per deposition cycle for films deposited by the immersion method on 11-mercapto-1-undecanol (MUD) and 16-mercaptohexadecanoic acid (MHDA), respectively. In contrast, the films deposited by the spray method are thicker with the same average thickness per deposition cycle (21 Å) for both SAMs. While the spray method takes less time to grow thicker films, it produces similar crystallite structures, regardless of the surface functionalization. This research is fundamental to understanding the impact of deposition method and surface functionalization on surMOF film growth and to provide strategies for the preparation of high-quality surMOFs.

## Introduction

Metal–organic frameworks (MOFs) are an important class of organic–inorganic hybrid supramolecular materials that consist of metal ions coordinated to organic ligands to form porous, highly crystalline structures.^1−3^ Owing to their unique properties and easily tunable structures, they attract great interest in a wide range of applications, including gas separation,^1^ storage,^4,5^ chemical sensing,^2^ and catalysis.^3^ Future applications, specifically in the fields of electronics and photonics,^6,7^ require the development of MOF thin-film deposition techniques for incorporation into device architectures. Films with tailored thickness, roughness, crystallographic orientation, and mechanical stability are highly desirable for the advancement of their applications. For the formation of MOF thin films, many deposition methods have been reported, including solvothermal,^8^ electrochemical,^9^ vapor phase,^10^ layer-by-layer (LBL),^11−13^ and drop-casting.^14^ The solution-phase LBL approach is a commonly used method to produce surface-anchored MOF (surMOF) thin films with sub-100 nm thicknesses.^12,15^

For surMOF deposition by the LBL approach, substrates are functionalized with self-assembled monolayers (SAMs) such that the terminal surface functional groups anchor the surMOF film and can direct film morphology, crystallographic orientation, and roughness.^16−20^ SurMOF films are LBL deposited by alternating immersion of these chemically modified substrates in solutions of the metal and organic components. Between metal and organic deposition steps, the substrates are rinsed by immersing in a solvent to remove excess unreacted starting materials on the surface. This immersion-based LBL method is a common approach that has been explored for the formation of an array of MOF systems; however, it requires moderate periods of time (hours) to complete and consumes large amounts of chemicals. To minimize these limitations, an LBL spray deposition method has been developed.^21−24^ This method is similar to the immersion-based approach in that the SAM-functionalized gold substrates are alternately sprayed with metal precursor and organic ligand solutions. Residual reactants on the substrates are removed by rinsing with a solvent in between metal and organic deposition steps. The key advantages of this spray method are reduced chemical and time consumption (minutes) that produce thicker films.^15,24−26^ Both the immersion- and spray-based LBL methodologies control film thickness by regulating the number of deposition cycles. Comparative studies for these two approaches are limited in the literature and are necessary in order to determine if design rules developed for the more common LBL immersion-based method translate to the spray deposition technique.

To incorporate surMOF systems into device architectures, the fundamentals of surMOF film formation must be explored and understood for a variety of MOF systems as well as for different deposition methods to further understand how film growth can be tailored to meet design requirements. The study herein builds on the few studies available in the literature regarding the formation of surMOFs examining the growth mechanism through a systematic investigation of their nanoscale surface features.^14,17,19,27−30^ This research investigates the growth of Cu-BDC (copper benzene-1,4-dicarboxylate) surMOF films using atomic force microscopy (AFM) complemented with powder X-ray diffraction (XRD), IR spectroscopy, and ellipsometry analysis. Cu-BDC was selected for this investigation because it is a well-studied MOF powder with applications for catalysis and sensing technology.^31−33^ This MOF system is also called Cu-MOF-2 because it is an analogue of MOF-2, which contains Zn. Cu-BDC is a paddle-wheel-based MOF system, where a dimer of copper(II) ions is equatorially coordinated to four carboxylate groups,^13,34^ with axial positions coordinated to water or solvent molecules that can be removed to activate the MOF for applications, such as gas separation, gas sorption, and catalysis.^14,35^ In addition, this MOF system has been deposited under ambient conditions using ethanol as a solvent to form a surMOF that is stable in water, which is a promising quality to develop them as effective antimicrobial coatings for life science applications.^36^ Cu-BDC exhibits a C2 symmetry when synthesized as a powder.^31,37^ However, when previously synthesized as a surMOF using the LBL method, a high-symmetry P4 structure was observed.^38,39^ Although some structural details of this material as a surMOF are reported throughout the literature,^36,38,39^ surface morphology characterized by high-quality AFM revealing the nanoscale features of the film has not been studied.

In the study, herein, a series of experiments were performed to systematically study the growth of Cu-BDC on gold substrates functionalized with either carboxyl- or hydroxyl-terminated alkanethiol SAMs. Alternating, sequential, solution-phase LBL immersion and spray deposition methods were used to deposit Cu-BDC surMOF films at room temperature. High-resolution AFM images were collected throughout the deposition process to understand the growth of Cu-BDC surMOF as a function of SAM composition and deposition method. Image analysis was conducted to determine the film roughness and surface coverage. Film crystallinity was investigated by powder X-ray diffractometry. Further characterization was undertaken by ellipsometry and IR spectroscopy to examine the film thickness and chemical composition, respectively. Water and thermal stability as well as adhesive properties of the surMOF film were tested in order to understand the impact of environmental conditions on film quality. Toward the development of high-quality surMOFs designed for targeted applications, this systematic study explores the comparison of LBL immersion and spray methods to deposit Cu-BDC surMOFs on different surface functionalities.

## Experimental Section

### Materials

For the formation of Cu-BDC films, 16-mercaptohexadecanoic acid [MHDA] (90%), 11-mercapto-1-undecanol [MUD] (97%), copper(II) acetate monohydrate [Cu(CH_3_COO)_2_·H_2_O] (≥98%), and terephthalic acid [H_2_BDC] (98%) were purchased from Sigma-Aldrich. Absolute, anhydrous ethanol (200 proof ACS/USP grade) was purchased from Pharmco by Greenfield Global. Gold-coated (100 nm) silicon wafers with an adhesive titanium layer (5 nm) were purchased from Platypus Technologies.

### Immersion-Based Layer-by-Layer Deposition of Cu-BDC Films

Cu-BDC films were deposited by the solution-phase layer-by-layer (LBL) immersion method on gold substrates functionalized with MHDA or MUD SAMs (self-assembled monolayers). The SAMs were formed by submerging the substrates in 1 mM ethanolic solutions of MHDA and MUD for 1 and 24 h, respectively. SAM-functionalized gold substrates were then rinsed with ethanol and dried under a flow of nitrogen. For Cu-BDC film deposition, ethanolic solutions of 1 mM Cu(CH_3_COO)_2_·H_2_O and 0.1 mM H_2_BDC were used to provide the metal and organic components. A Midas III-Plus Automated Slide Stainer was employed to deposit metal and organic components in an alternating fashion. One deposition cycle includes 30 min metal deposition, 5 min ethanol rinse, 10 min dry at 30 °C, 1 h organic deposition, 5 min ethanol rinse, and 10 min dry at 30 °C. These steps were repeated for 4, 8, 12, 16, and 20 deposition cycles. Deposited films were characterized by ellipsometry, atomic force microscopy (AFM), powder X-ray diffractometry (XRD), and infrared spectroscopy (IR) to determine the film thickness, morphology, crystallinity, and functional groups, respectively. Samples were then stored in a dry box for further analysis.

### Stability Experiments

Experiments to test for water and thermal stabilities as well as adhesive properties were performed for samples with Cu-BDC films formed by 20 LBL immersion deposition cycles (20*L*) deposited on MHDA or MUD SAMs. Before and after each stability experiment, the films were characterized by ellipsometry, XRD, and AFM. To investigate water stability, samples were immersed in deionized water for 1 min as well as 1 h, rinsed with ethanol, and dried using nitrogen gas. Thermal stability was tested by placing films under high vacuum with heating to 110 °C for 1 h. A tape test was performed to determine the adhesive properties of the films. To perform the tape test, a transparent piece of Scotch tape was placed over the film, pressed gently with a finger, and peeled off immediately afterward.

### Layer-by-Layer Spray Deposition of Cu-BDC Films

For spray deposition of Cu-BDC, substrates were mounted on a sample holder and subsequently sprayed with ethanolic solutions of metal and organic linkers (1 mM Cu(CH_3_COO)_2_·H_2_O and 0.1 mM H_2_BDC). Working air pressure was 60 psi (additional spray deposition details are available in the Supporting Information). One deposition cycle includes 20 s metal deposition, ethanol rinse, dry under a nitrogen flow, 20 s organic deposition, ethanol rinse, and dry under a nitrogen flow. A series of five samples were routinely prepared on both MHDA- and MUD-functionalized gold substrates at room temperature. Samples were sprayed with 4, 8, 12, 16, and 20 deposition cycles. Samples were stored in a dry box for further analysis. Ellipsometry, AFM, and XRD were used as characterization techniques to analyze film properties.

### Atomic Force Microscopy (AFM)

Film morphology was imaged using a Park Systems NX10 AFM with a PPP-NCH 10 M probe (42 N/m force constant) in noncontact mode. SmartScan operating software was employed to collect four 2.5 μm × 2.5 μm images (256 × 256 pixels) at four different locations for each sample. Scan parameters were 1 Hz scan rate and 12 nm set point using an XY scanner with a single module flexure, closed control, and a scan range of 50 μm × 50 μm. Film morphology and surface roughness (*R*_q_) were analyzed using XEI data processing and analysis software (Park Systems). ImageJ analysis was used to calculate the particle surface coverage. Average roughness and surface coverage values along with standard deviations were calculated by considering at least three sample replicates and a minimum of three spots per sample.

### Ellipsometry

Ellipsometry measurements were conducted using a single-wavelength, fixed-angle LSE Stokes ellipsometer (Gaertner Scientific Corporation). Film thickness data were acquired using a helium–neon laser with a wavelength of 6328 Å at an incidence angle of 70°. Film thickness was calculated using GEMP analysis software. The fixed values of 1.5 and 0 were used as the index of refraction (*n*_f_) and the extinction coefficient (*k*_f_), respectively.^27,40,41^ Samples were analyzed before and after SAM formation and then after each set of four Cu-BDC deposition cycles. A minimum of six spots per sample were collected for LBL-deposited samples, and three spots per sample were collected for spray samples. Average thickness and standard deviation values presented are representative of at least three replicates for each sample type.

### Powder X-ray Diffraction (XRD)

XRD patterns of Cu-BDC films were collected at room temperature using a Rigaku Miniflex II benchtop diffractometer operated at 30 kV and 15 mA with Cu Kα radiation (λ = 1.5418 Å). Diffraction patterns were collected using a sampling width of 0.03° and a scan speed of 1.000 or 0.500° per minute. Patterns were recorded in the 7–18° 2θ range to observe out-of-plane XRD peaks of Cu-BDC, which appeared around 9 and 17° 2θ.^38^

### Infrared Spectroscopy (IR)

A PerkinElmer IR spectrometer was used to collect IR spectra of Cu-BDC films from 4000 to 600 cm^–1^ in attenuated total reflection (ATR) mode. Spectra were collected over a set of 64 scans at a resolution of 4 cm^–1^. An unmodified gold substrate was used as the background.

## Results and Discussion

Solution-phase LBL immersion and spray methods were employed to deposit Cu-BDC surMOF films on gold substrates functionalized with either carboxyl- or hydroxyl-terminated alkanethiol self-assembled monolayers (SAMs). A series of samples were prepared with 4, 8, 12, 16, and 20 LBL deposition cycles (*L*). To investigate the effect of SAM composition and deposition method on film growth, films were characterized after every four deposition cycles by atomic force microscopy (AFM), ellipsometry, X-ray diffractometry (XRD), and infrared spectroscopy (IR). AFM is used to analyze the morphological features of films. Thickness values were calculated using ellipsometry after SAM formation and surMOF deposition. XRD is employed to investigate crystallinity, and IR is used to confirm the presence of functional groups. To further understand film properties, water and thermal stabilities as well as adhesion characteristics of the films were tested.

### Cu-BDC surMOF Films Characterized by the LBL Immersion Method

**Figure 1:**
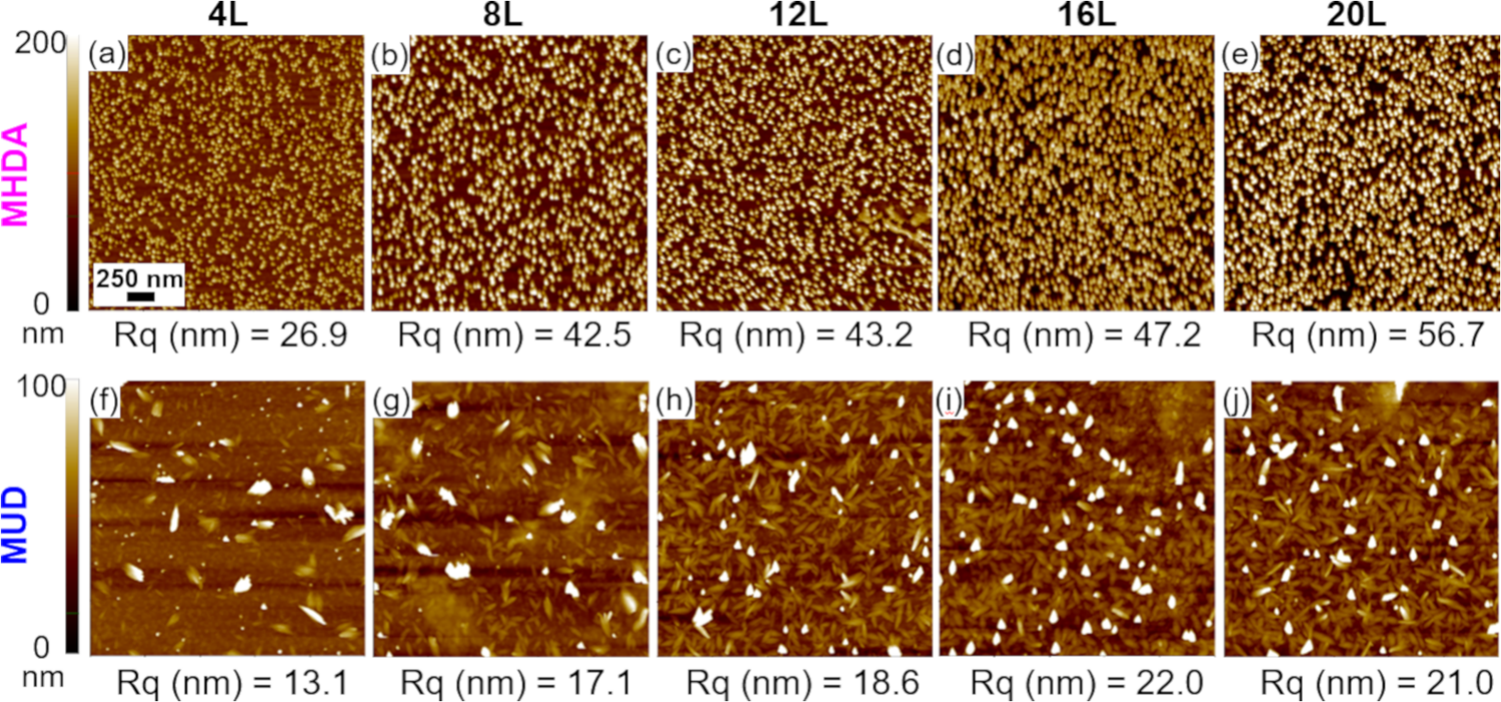
Representative atomic force microscopy images (2.5 μm × 2.5 μm) of Cu-BDC films deposited using the layer-by-layer (LBL) immersion method on Au substrates functionalized with (a–e) carboxyl-terminated MHDA (16-mercaptohexadecanoic acid) and (f–j) hydroxyl-terminated MUD (11-mercapto-1-undecanol) SAMs. Above each image, the number of LBL deposition cycles (*L*) completed prior to analysis is given. Below each image, the corresponding surface roughness value (*R*_q_) is provided and is specific to the AFM image. Scale bar of 250 nm (a) is for all images. In each row, all of the images are set to the same *z*-scale shown to the left of images (a) and (f).

**Figure 2:**
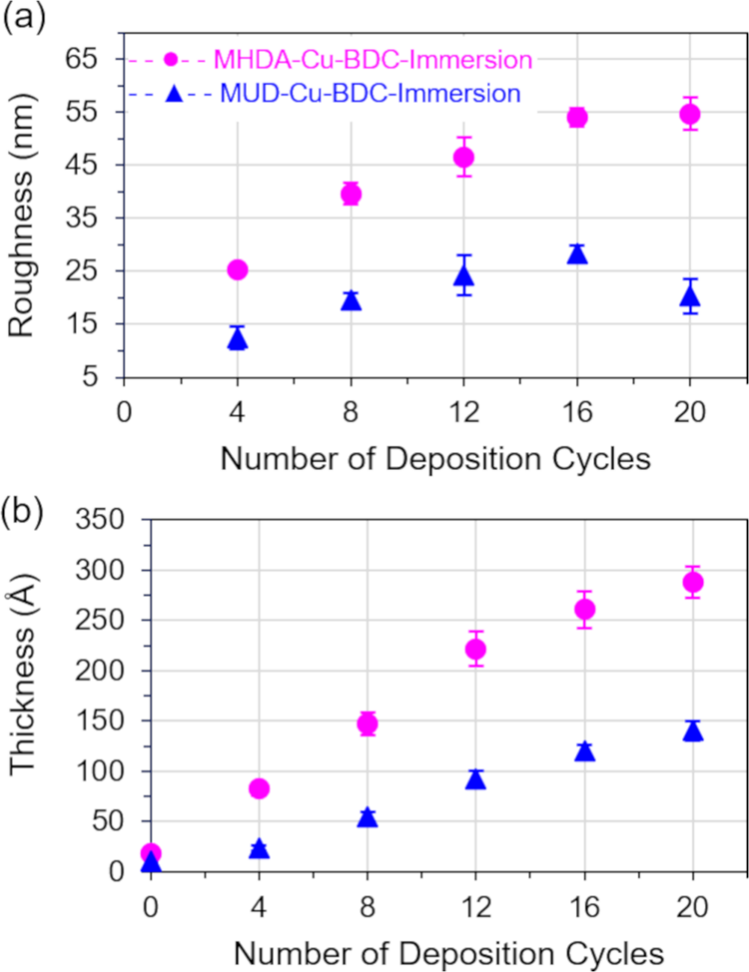
Films were deposited by the LBL immersion method on gold substrates functionalized with MHDA (pink circles) and MUD (blue triangles) SAMs. (a) Surface roughness (*R*_q_) of Cu-BDC films was determined by AFM. (b) Film thickness was determined by ellipsometry. Average roughness, average thickness, and corresponding standard deviation values are plotted as a function of deposition cycles.

AFM images and associated surface roughness values for Cu-BDC surMOF films deposited on SAM-functionalized gold substrates are shown in Figure 1. Films deposited on carboxyl-terminated MHDA are shown in the top row of images found in Figure 1a–e. Crystallite formation, consistent with a Volmer Weber growth mechanism, is observed after 4 deposition cycles. Film roughness (Figure 2a), film thickness (Figure 2b), and surface coverage (Figure S1) increase for the growth of Cu-BDC on MHDA SAM with increasing deposition cycles. A small increase in the size of the particles is observed in the 8*L* image compared to the 4*L*, however, no significant increase in the width of the particles is observed upon subsequent film deposition. Film morphology remains consistent throughout the series. The increase in film thickness and roughness without a significant change in particle width (∼50 nm) or morphology suggests that the crystallites are primarily growing vertically to the sample as nanorods or nanowires.

SurMOF films deposited on hydroxyl-terminated MUD are shown in the bottom row of Figure 1f–j. Note that these images are set to a lower *z*-scale compared to MHDA samples to clearly observe the surface features. A Volmer Weber film formation is observed for samples deposited on MUD-coated substrates similar to MHDA samples. However, less crystallite growth is observed on MUD at the initial stages with the number of particles on the surface increasing with additional deposition cycles. In contrast to MHDA samples with crystallites of uniform size and shape that are presumed to be vertical rods, films of Cu-BDC deposited on MUD are primarily composed of lying-down rod-shaped crystallites. Films deposited on MUD have lower surface roughness values (Figure 2a) and lower film thicknesses (Figure 2b) compared to the samples deposited on MHDA. These smoother and thinner films are consistent with the different lying-down crystallite growth observed by AFM for Cu-BDC on MUD SAMs. SAMs with different functional groups can act as templates for oriented crystal growth by altering the anchoring ability of metal and organic linkers to the surface. This has been reported in the literature for other MOF materials deposited on MHDA and MUD SAMs.^16−20^ The carboxyl-terminated functional groups for MHDA SAMs mimic the organic linker of the MOF coordinated equatorially to the copper paddle-wheel inorganic node, whereas hydroxyl-terminated groups for MUD SAMs imitate coordinated water molecules typically bound at the axial position of the copper paddle-wheel unit. This difference in the terminal functional group of the SAM alters how the copper dimers bind to the substrate and impact the crystal growth direction.^25,42^

Film thickness and associated standard deviations are shown in Figure 2b for samples deposited on MHDA and MUD SAMs. The thickest films are obtained after 20 deposition cycles and have thicknesses of approximately 30 and 15 nm for samples deposited on MHDA and MUD SAMs, respectively. Linear film growth is observed for both samples, and linear fits for the ellipsometric data (Figure S2a) reveal slopes of 1.4 and 0.70 nm per deposition cycle for films on MHDA and MUD samples, respectively. This result is consistent with the morphological difference observed in AFM for samples deposited on MHDA and MUD SAMs.

**Figure 3:**
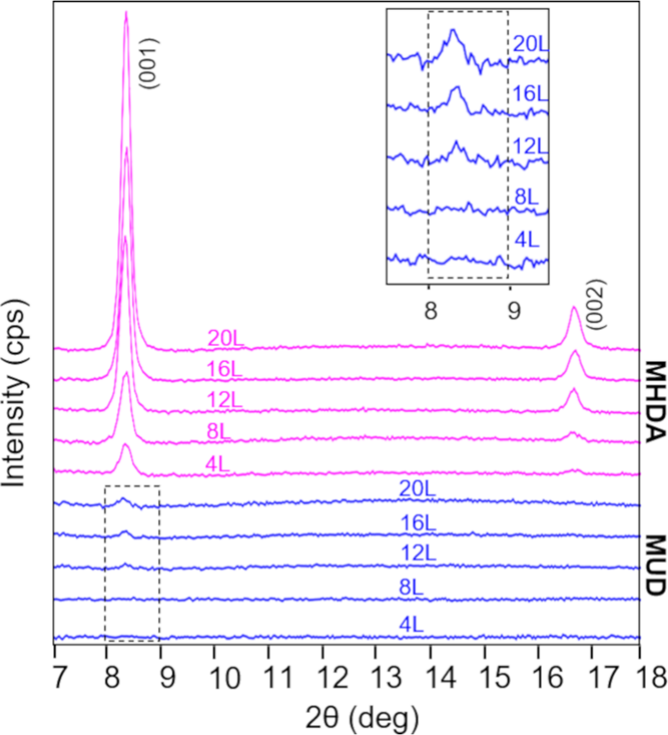
XRD patterns of Cu-BDC films deposited by the LBL immersion method on MHDA (pink, top set) and MUD (blue, bottom set) SAMs. Inset shows the XRD peaks of MOF-2 deposited on MUD SAM in the 8–9 2θ range. The number of LBL deposition cycles (*L*) is represented above the corresponding pattern.

Figure 3 shows the XRD patterns collected for the films deposited on MHDA and MUD after 4, 8, 12, 16, and 20 deposition cycles. For these Cu-BDC surMOFs deposited by the LBL immersion method, characteristic XRD peaks for the Cu-BDC MOF appear at 2θ values of ∼9 and ∼17°, corresponding to the (001) and (002) crystalline planes, respectively.^38,39^ These characteristic peaks are clearly visible in all five samples deposited on MHDA supporting the crystalline nature of the Cu-BDC surMOF films. Increasing peak intensity is observed with increasing deposition cycles, demonstrating the presence of more material on the surface. These XRD patterns are consistent with the patterns previously obtained for Cu-BDC films fabricated on gold substrates functionalized with MHDA.^38,39^ In contrast to MOF powders, the deposited films show no lateral shift between stacks of copper paddle-wheel planes, resulting in a high-symmetry P4 structure with a simple tetragonal unit cell.^38,39^ For the thinner films deposited on MUD, the XRD peak at 2θ = 8.5° was observed for films fabricated by 12, 16, and 20 deposition cycles. From the 12 to 20 deposition cycles, the intensity of this peak is observed to increase (Figure 3 inset). The intense XRD peak associated with the surMOF film on the MHDA sample correlates with a high degree of preferred orientation consistent with the standing-up nanorod crystallites suggested by the AFM data (Figure 1a–e). The less intense XRD peaks for surMOF films on MUD samples are due to the absence of long-range crystalline order for samples deposited on MUD, as shown by randomly oriented lying-down nanorod crystallites seen in the AFM images (Figure 1f–j).

**Figure 4:**
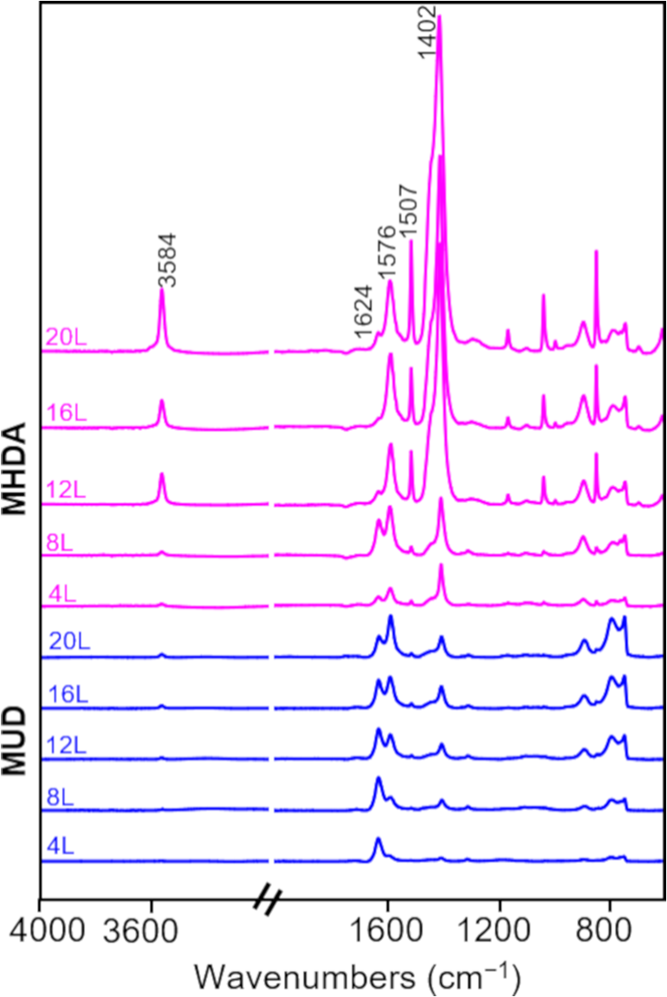
Representative infrared spectra for Cu-BDC films deposited by the LBL immersion method on MHDA (pink, top set) and MUD (blue, bottom set) SAMs. The number of LBL deposition cycles (*L*) is represented above the corresponding spectrum.

To further understand the chemical composition and the film growth, samples were analyzed by IR spectroscopy after every four deposition cycles. IR spectra collected for Cu-BDC films deposited on MHDA and MUD are shown in Figures 4 and S3. Characteristic IR peaks for Cu-BDC are detected in all samples.^13,43,44^ The IR peaks at 1624 and 1402 cm^–1^ wavenumbers correspond to asymmetric and symmetric vibrations of the carboxylate group, respectively. The presence of an aromatic ring is demonstrated by the appearance of peaks at 1576 and 1507 cm^–1^, which correspond to the C–C stretches in the aromatic ring.^43,44^ A sharp peak is detected in most of the samples at 3584 cm^–1^, which is associated with O–H stretching. The appearance of this sharp peak suggests the presence of −OH groups that are not hydrogen bonded to other molecules.^32,44^ As shown in Figure 4, the main differences in the IR spectra of the samples deposited on MHDA and MUD are the intensity ratio of the peaks at 1576 and 1507 cm^–1^ and the intensity ratio of the peaks at 1624 and 1402 cm^–1^. It is postulated that these differences likely arise due to the difference in growth orientation of surMOF crystallites.^42^

The deposition times of 1 h for MHDA and 24 h for MUD were selected to optimize SAM density and surMOF film quality (Figures S4 and S5). When surMOFs were deposited on MHDA SAMs that had been assembled for 24 h, AFM data showed that the uniformity of the resulting surMOF vertical nanorod crystals was less than observed for the MHDA SAM assembled in 1 h (Figure S4). Ellipsometry data showed that the MHDA SAM assembled over a 24 h time period was approximately twice as thick as the SAM formed over 1 h, which is likely due to the formation of a bilayer with carboxylic acids hydrogen bonded in dimers. Additionally, ellipsometry showed that the surMOF deposition on the 24 h MHDA SAM was less than that on the 1 h MHDA SAM (Figure S5a). Thus, to enhance MHDA SAM quality and surMOF uniformity, 1 h assembly for the MHDA SAM was selected. Additionally, XRD characterization did not reveal significant differences for surMOF films anchored to an MHDA SAM assembled for 1 h versus 24 h (Figure S5b), which is consistent with the AFM data, showing that the majority of the crystallites were oriented vertically in both cases. When SAMs of 1 h MUD were compared to 24 h MUD SAMs, no significant differences were observed by AFM, ellipsometry, or XRD (Figures S4 and S5). Therefore, to produce a dense and well-ordered film of the shorter alkane chain MUD SAM, 24 h was selected for this systematic study.

### Film Adhesion and Stability

**Figure 5:**
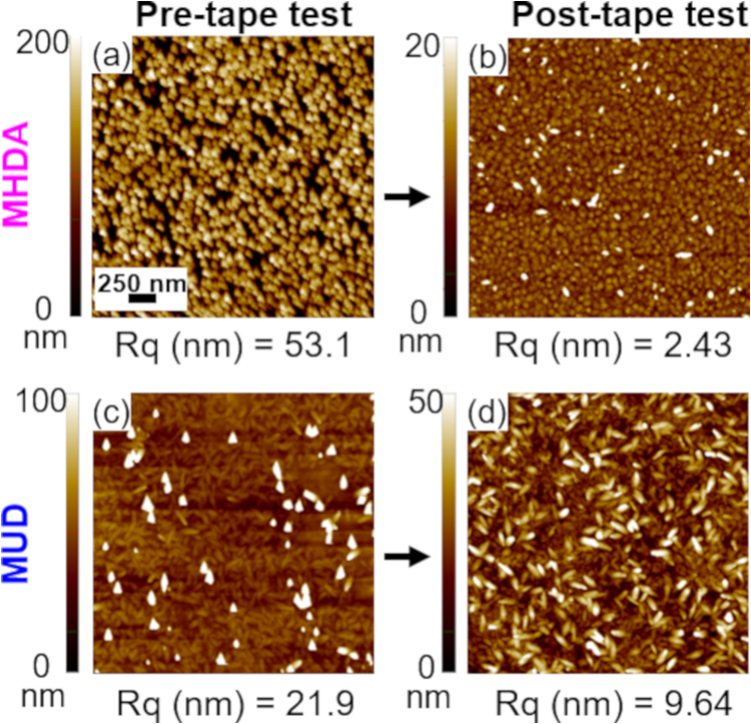
Representative atomic force microscopy images (2.5 μm × 2.5 μm) of Cu-BDC films deposited by 20 LBL immersion deposition cycles. Images are collected before (a, c) and after (b, d) tape test for (a, b) surMOF films on MHDA SAMs and (c, d) on MUD SAM. Below each image, the corresponding surface roughness (*R*_q_) is given.

Toward the integration of these surMOFs into industrial and specialized applications, the film robustness needs to be investigated with regard to adhesion as well as water and thermal stability. Adhesive properties of the Cu-BDC films deposited on MHDA and MUD SAMs are investigated by the tape test. AFM images in Figure 5 show the morphology of Cu-BDC films before and after the tape test. As seen in Figure 5a,b, films deposited on MHDA exhibit poor substrate adhesion as most crystals are removed from the surface by the adhered tape. Before the tape test, the substrate contained an abundance of Cu-BDC crystallites (Figure 5a), but after the tape is removed, the AFM images show the “cobblestone-like” grain structure of the gold substrate with just a few remaining crystallite protrusions (Figure 5b). Film roughness is reduced from 53 to 2.4 nm, which is slightly higher than the roughness of plain gold (∼1.5 nm), demonstrating that the vast majority of the standing-up nanorod crystallites were lifted-off from the substrate. This could be exploited by transfer lithography methods to manipulate these well-oriented nanocrystallites onto different substrates or into alternative structures. This is consistent with XRD data shown in Figure S6a, where the peak intensity is significantly decreased after the tape test. Films deposited on MUD, however, show a strong adhesion to the substrate as most crystals are still attached to the surface after the tape test (Figure 5c,d). The tape removed most of the tall Cu-BDC crystallites deposited on MUD, but lying-down rod structures remain intact. Roughness values decrease from 22 to 9.6 nm due to removal of the tall features. These AFM results for surMOFs on MHDA and MUD are consistent with the ellipsometry thickness values calculated before and after the tape test (Table S1). Note for the film on MUD, no change in thickness was observed; however, the standard deviation was decreased due to the removal of standing-up crystallites. Better adhesion of Cu-BDC crystallites deposited on MUD compared to the crystals deposited on MHDA is likely due to high surface area coordination with the anchoring SAM for the lying-down crystals to the surface.

To understand the performance of the film properties under different environmental conditions, the susceptibility of the Cu-BDC films toward water (immersion in water for 1 min and 1 h) and heat (110 °C under vacuum for 1 h) was tested. Samples were analyzed by ellipsometry, AFM, and XRD before and after each stability experiment. Ellipsometry data (Table S1) and AFM images (Figure S7) illustrate that film thickness, surface coverage, and film morphology remain unchanged for the films deposited on MHDA, regardless of the experimental conditions. Cu-BDC on MUD has deposited crystallites on the substrate after stability experiments (Figure S7g–i), but the number and size of particles on the surface differ depending on the experimental condition. After 1 h in water, the Cu-BDC on the MUD sample has fewer standing-up particles and a decrease in film roughness compared to the 1 min and control sample. Also, particle morphology for the Cu-BDC on MUD samples changed slightly after the thermal stability experiment with fewer and larger lying-down nanorods covering the surface. Ellipsometry data for Cu-BDC deposited on MUD does not show significant changes in the film thickness with all thicknesses determined after stability experiments being within the error of the as-deposited film (Table S1). XRD patterns for the Cu-BDC films deposited on MHDA and MUD after stability experiments are plotted in Figure S6. The data reveal no changes in peak positions when compared to XRD patterns for the as-deposited surMOFs, confirming the crystalline stability of the samples under different environmental conditions. AFM, ellipsometry, and XRD data indicate that the Cu-BDC surMOF films on MHDA and MUD are thermally stable when placed in a vacuum oven for 1 h at 110 °C. According to the literature reported thermogravimetric analysis data, the Cu-BDC powder is thermally stable up to 300 °C.^37,45^ Moreover, Cu-BDC surMOF films are stable when immersed in water for 1 h, which is consistent with literature precedent.^36^

### Deposition of surMOF Films Using the Spray Method

**Figure 6:**
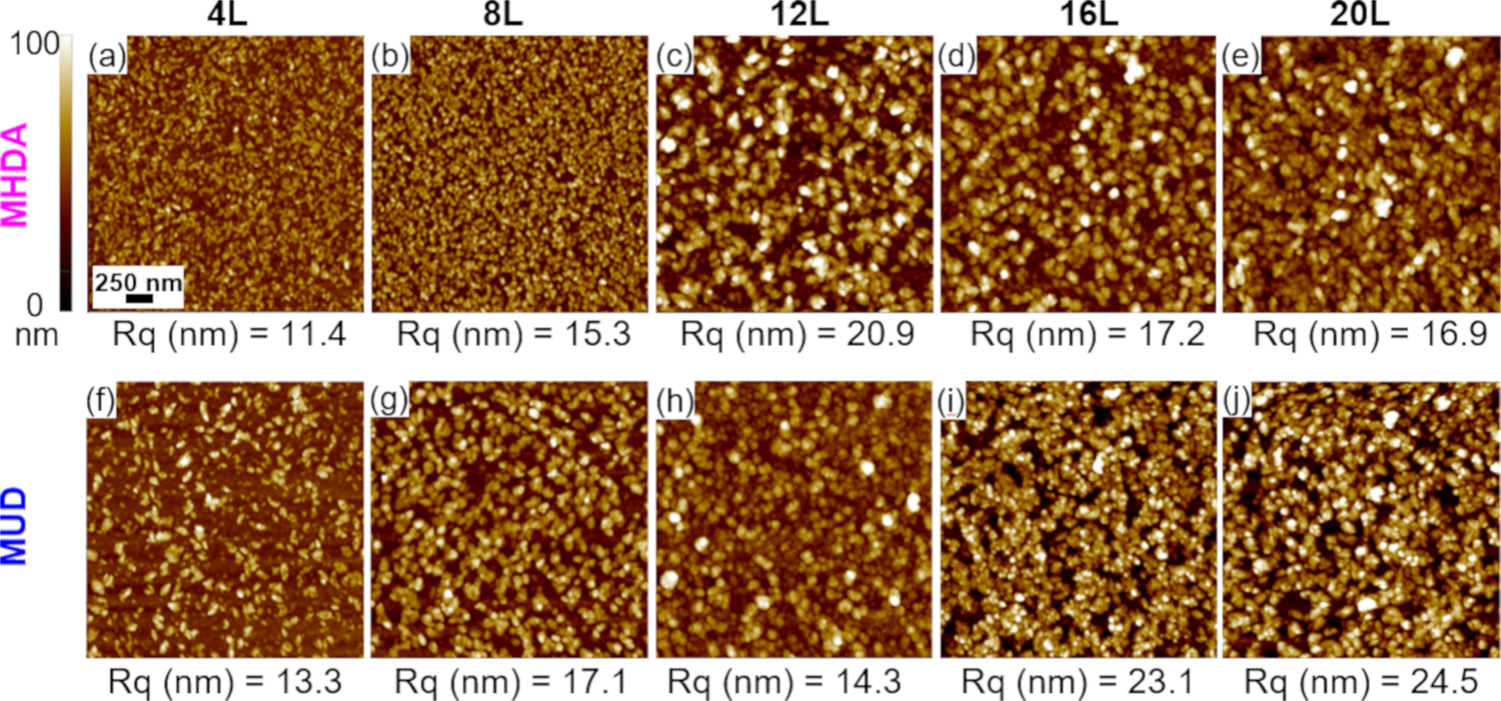
Representative atomic force microscopy images (2.5 μm × 2.5 μm) of Cu-BDC films deposited using the LBL spray method on Au substrates functionalized with (a–e) MHDA and (f–j) MUD SAMs. Above each image, the number of LBL deposition cycles (*L*) completed prior to analysis is given. Below each image, the corresponding surface roughness value (*R*_q_) is provided and is specific to the AFM image. Scale bar of 250 nm (a) is for all of the images. All of the images are set to the same *z*-scale.

**Figure 7:**
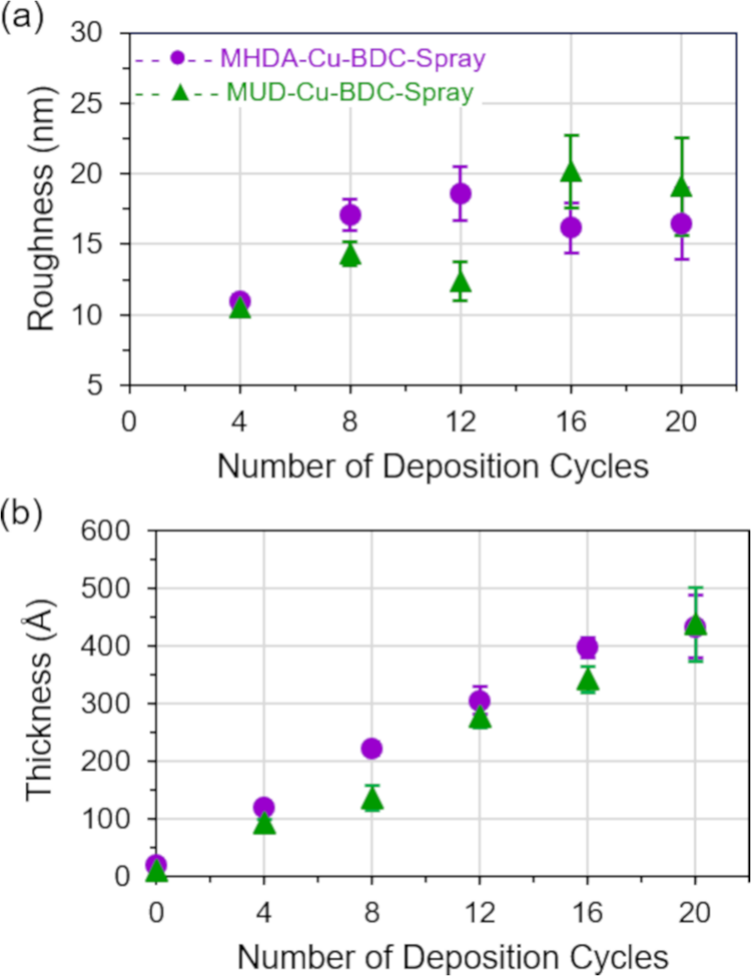
Films were deposited by the LBL spray method on gold substrates functionalized with MHDA (purple circles) and MUD (green triangles) SAMs. (a) Surface roughness (*R*_q_) of Cu-BDC films was determined by AFM. (b) Film thickness was determined by ellipsometry. Average roughness, average thickness, and corresponding standard deviation values are plotted as a function of deposition cycles.

Cu-BDC films were also deposited by the LBL spray deposition method to understand the impact of deposition method on the growth. Similar to immersion-based LBL deposition, the spray method deposited surMOF films on SAM-functionalized substrates by alternately spraying the substrate with metal and organic linker solutions followed by ethanol rinsing and drying under nitrogen. Figure 6 shows the representative AFM images of the films deposited on MHDA and MUD SAMs on gold substrates. For the surMOF films deposited on MHDA (Figure 6a–e), the surface coverage and particle sizes are similar for the 4*L* and 8*L* samples. After subsequent deposition cycles, particle size increases and crystallites are less uniform in shape. The highest surface roughness values are observed for the 12*L* sample (Figure 7a) with a slight decrease in roughness observed thereafter. For the Cu-BDC surMOFs deposited on MUD, irregular shape and size are observed after 4*L* and continue throughout film deposition. In contrast to the LBL immersion method, films deposited using the spray method do not show rod-like crystallites and a difference in crystallite orientation is not observed by AFM depending on the surface functionalization. The film growth for the Cu-BDC surMOF is significantly impacted by this different method of deposition.

Film thicknesses measured by ellipsometry are shown in Figure 7b. Films deposited on both MHDA and MUD have similar thickness values based on the number of deposition cycles with the highest thickness values being 45 nm after 20 deposition cycles. This film growth is linear and a linear fit shows a slope of 2.1 nm for both surMOFs on MHDA and MUD (Figure S2). These values are 1.5 and 3 times greater than the thicknesses of Cu-BDC deposited by the LBL immersion method on MHDA and MUD SAMs, respectively. While the spray method is an efficient technique to obtain thicker films, the film structures formed do not appear to be influenced by the terminal functional group of the SAM coating on the substrate.

Spray-deposited samples were further analyzed using XRD to explore the crystallinity of the films deposited on MHDA and MUD SAMs (Figure S8). All five samples deposited on MHDA show characteristic peaks at 2θ = 8.5° (001) and 17° (002), consistent with the structure of Cu-BDC. The appearance of these two peaks is less distinct for the surMOF films deposited on MUD despite their comparable thicknesses to samples deposited on MHDA. This suggests that while there is no significant difference in the film morphology observed by AFM, there is likely a difference in the quality and size of the crystallite grains.

The tape test was also performed for 16*L* and 20*L* Cu-BDC films deposited by the LBL spray method on MHDA and MUD SAMs. Film thicknesses obtained for surMOFs on MHDA and MUD SAMs before and after the experiment are consistent within the standard deviation of the as-deposited films (Table S2). This data shows that there are strong adhesive properties for films deposited by the LBL spray method on both the MHDA and MUD SAMs. This observation is different than the tape test results for the films deposited by the LBL immersion method on MHDA SAM (Table S1).

### Comparison of Deposition Methods

Film morphologies for Cu-BDC surMOFs were different depending on the deposition method with distinct and uniform crystallite structures observed for films deposited by the LBL immersion method (Figure 1) and more irregularly shaped crystallites observed for surMOFs deposited by the LBL spray method (Figure 6). For films deposited by the immersion method, the terminal functional group of the anchoring SAM directed the orientation of the crystallite growth yielding standing-up nanorods on MHDA and lying-down nanorods on MUD, resulting in distinctly different film roughness and thickness values (Figure 2). AFM topographic mapping permits the resolution and visualization of these nanoscale MOF structures beyond the capabilities of conventional scanning electron microscopy (SEM) (Figure S9). Future research utilizing field-emission SEM may provide high-resolution data to further investigate surMOF crystal size and morphology without AFM tip-sample convolution. Thicker films were observed by ellipsometry for the spray method, but there was no difference in film roughness or thickness values between the surMOF films deposited by this method on MHDA and MUD SAMs (Figure 7). Crystallinity consistent with Cu-BDC surMOFs was indicated for both methods, however, the highest intensity of these peaks was observed for films on MHDA, while less intense peaks were found for films deposited on MUD.

The observations and measurements collected in this study suggest differences in the formation of the surMOF film for these two deposition techniques. For samples fabricated by the LBL immersion method, oriented crystallite growth is observed with differences in the terminal functional group of the anchoring SAM producing a remarkable morphological difference for the Cu-BDC surMOF. As reported in the literature for the other surMOF systems, the presence of solvents promotes a reversible coordination bonding between metal ions and organic ligands, facilitating a self-repairing process during the deposition.^24^ In the immersion method, samples are immersed in solutions during the deposition, thus providing time for the self-repairing process. However, in the spray method, this restructuring of particles is restricted due to less contact time between the substrate and solvent, resulting in limited mobility of the MOF components at the substrate–solvent interface. This reduced mobility may be the variable that results in less oriented film growth on the substrate.

Ellipsometric measurements show that the thickness of surMOF films deposited by the immersion method is less than that obtained for the spray method (Figures 2b, 7b, and S2). The larger thickness obtained by the spray method is consistent with other research investigating spray deposition and is postulated to be due to the less effective rinsing during the deposition process.^21^ In the immersion method, samples are rinsed with ethanol using agitation to remove loosely bound crystallites and any excess precursor. This contrasts with the rapid rinsing step in the spray method, which uses a smaller volume of solvent that may not be sufficient to remove all excess materials from the substrate. Another potential mechanism is that unremoved starting materials could be stored in the pores of already deposited MOF as well as on the surface of the film, thus creating a thicker film than the immersion method.^46^

As compared in Figure S2, Cu-BDC films deposited by the immersion method increased by 1.4 nm per deposition cycle on MHDA and 0.70 nm per deposition cycle on MUD. Thickness of the films deposited using the spray method increased by 2.1 per deposition cycle independent of underlying SAM. The size of the organic linker, ∼0.7 nm, correlates with these increases in film thickness such that one and two layers are deposited per immersion deposition cycle on MUD and MHDA, respectively, and three are deposited per spray deposition cycle.

## Conclusions

This research investigated the effects of deposition method and SAM composition on Cu-BDC surMOF growth with a series of samples prepared using LBL immersion and spray methods on gold substrates functionalized with carboxyl- (MHDA) and hydroxyl- (MUD) terminated SAMs. Using the LBL immersion method, surface functionalization was found to impact the morphology, crystallite orientation, surface roughness, surface coverage, and thickness of the Cu-BDC surMOF deposited. As elucidated by AFM, the nature of the functional group directed distinctly different surMOF crystallite orientations with MHDA SAMs anchoring rod-shaped crystals perpendicular to the surface and MUD anchoring rod-shaped crystals horizontal to the surface. This standing-up versus lying-down orientation resulted in the Cu-BDC surMOF on MHDA having larger roughness values. XRD confirmed crystallinity of the surMOF for both SAMs with much greater peak intensities observed for the films deposited on MHDA SAMs, revealing a significant preferred orientation. Additionally, films deposited by the LBL immersion method on MHDA were thicker when compared to the films on the MUD-functionalized substrates.

Stability experiments were performed to understand the impact of environmental conditions on film quality. For the surMOFs deposited by the LBL immersion method, a tape test elucidated that the lying-down rod structures on MUD SAM were adherent to the substrate, whereas the majority of standing-up crystallites on MHDA SAM were removed. Ellipsometry, AFM, and XRD showed that the quality of the Cu-BDC films was maintained after water and thermal stability experiments for films on both MHDA- and MUD-functionalized substrates.

Films deposited by the LBL spray method on MHDA and MUD SAMs had the same morphology, surface roughness, crystallite orientation, and film thickness. The LBL spray method is desirable in that it reduces time and material consumption. However, the findings herein show that it inhibits the influence of chemical functional groups on SAMs to control the film formation. Films deposited by LBL spray were thicker than those formed by LBL immersion. This increased thickness is likely due to the reduced influence of the SAM and a different rinsing procedure (shorter time without agitation). In the LBL immersion method, the substrate is maintained in the solution for a prolonged time period that permits equilibrium to be reached and reorganization of surMOF components, such that the average thickness per deposition cycle for MUD and MHDA was 0.70 and 1.4 nm, respectively. For the LBL spray method, the thickness per layer is the same (2.1 nm) for both systems reproducibly. This is likely dependent on spray deposition conditions, such as solution concentration, spray time, rinsing method, and drying procedure. Future research will explore how these reaction conditions impact the Cu-BDC surMOF growth. These films formed by spray on MHDA and MUD SAMs displayed adhesive properties similar to the films deposited by LBL immersion on MUD but had their own unique morphology with irregularly shaped nanocrystallites.

Overall, for this Cu-BDC surMOF system, surface functionalization is found to influence the film morphology for immersion-based deposition but does not affect film formation for spray deposition. Future research will investigate different surface functional groups and MOF systems. While LBL immersion is more time and material consuming, it offers more control over the film morphology using surface functionalization. This is advantageous for applications that require highly oriented ultrathin films. This study highlights how the different deposition methods and surface functional groups change the film qualities of Cu-BDC surMOF. Research presented in this study will benefit researchers that seek to design and fabricate high-quality surMOF films for applications ranging from gas storage, sequestration, and separation to electronic, photonic, and sensing technologies.

## Supporting Information Available

Graph of percent surface coverage with ImageJ data analysis procedure; full IR spectra; ellipsometric, AFM, and XRD data associated with the LBL immersion method on 24 h MHDA and 1 h MUD; experimental procedure for stability tests and associated ellipsometric, AFM, and XRD data; linear fits of ellipsometric thickness data; experimental procedure for spray deposition; XRD patterns of spray-deposited samples; experimental procedure for scanning electron microscopy and associated images (PDF)
