# Supplementary material for: Impact of Surface Functionalization and Deposition Method on Cu-BDC surMOF Formation, Morphology, Crystallinity, and Stability
Source: Langmuir. 2023 Aug 16;39(34):12196–205. doi: 10.1021/acs.langmuir.3c01505 (PMC10469448; doi:10.1021/acs.langmuir.3c01505)
Supplement: Supplementary file 1 — la3c01505_si_001.pdf [file la3c01505_si_001.pdf]

Supplementary Information

Impact of Surface Functionalization and Deposition Method on Cu-BDC surMOF Formation, Morphology, Crystallinity, and Stability

B. Dulani Dhanapala, Dayton L. Maglich, Mary E. Anderson*

Department of Chemistry, Furman University, Greenville, South Carolina, USA

*Corresponding Author. Email: maryelizabeth.anderson@furman.edu

Included:

Surface coverage analysis using ImageJ. (Page S3)

Figure S1: Surface coverage of Cu-BDC films deposited by LBL immersion method. (Page S3)

Figure S2: Comparison of ellipsometric film thicknesses. (Page S4)

IR spectra. (Page S5)

Figure S3: IR spectra for films deposited by LBL immersion method. (Page S5)

Figure S4: AFM images of the films deposited using LBL immersion method on MHDA and MUD SAMs fabricated at 24 hr and 1 hr, respectively. (Page S6)

Figure S5: Comparison of thickness and XRD patterns for films deposited using LBL immersion method on MHDA and MUD SAMs fabricated at 24 hr and 1 hr, respectively. (Page S6)

Stability experiments. (Page S7)

Figure S6: XRD patterns of the films deposited by LBL immersion method before and after stability experiments. (Page S8)

Table S1: Ellipsometric thicknesses for the films deposited by LBL immersion method before and after stability experiments. (Page S8)

Figure S7: AFM images of the films deposited by LBL immersion method before and after stability experiments. (Page S9)

LBL spray deposition. (Page S10)

Figure S8: XRD patterns of the films deposited by LBL spray method. (Page S10)

Table S2: Ellipsometric thicknesses for the films deposited by LBL spray method before and after stability experiments. (Page S11)

Scanning electron microscopy (SEM). (Page S12)

Figure S9: AFM and SEM images of the films deposited by LBL immersion and spray method. (Page S13)

References. (Page 14)

Surface coverage analysis using ImageJ.

Collected AFM images were analyzed in the ImageJ program. To perform the analysis, images were converted to gray scale by changing the image type to 8-bit. Then, the images were adjusted to threshold by setting brightness to include all the particles on the sample. Surface coverage for each sample was obtained by selecting “analyze particles”. Surface coverage was calculated for at least three 2.5 × 2.5 μm AFM images from each deposition cycle in each sample set and there were three sample replicates. The obtained data was then compiled to determine the average and standard deviation for the surface coverage associated with each deposition cycle. These data are provided in Figure S1.

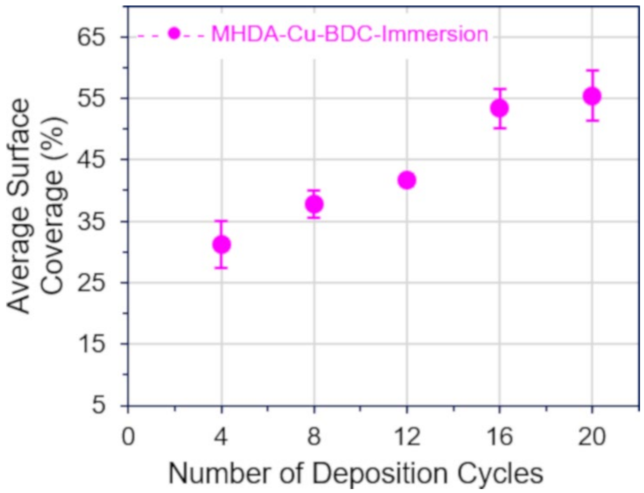

Figure S1. Surface coverage of Cu-BDC films deposited using LBL immersion method on MHDA SAMs as a function of number of deposition cycles. Average surface coverage and standard deviations are determined for 2.5 μm × 2.5 μm images using ImageJ analysis.

Comparison of ellipsometric film thicknesses.

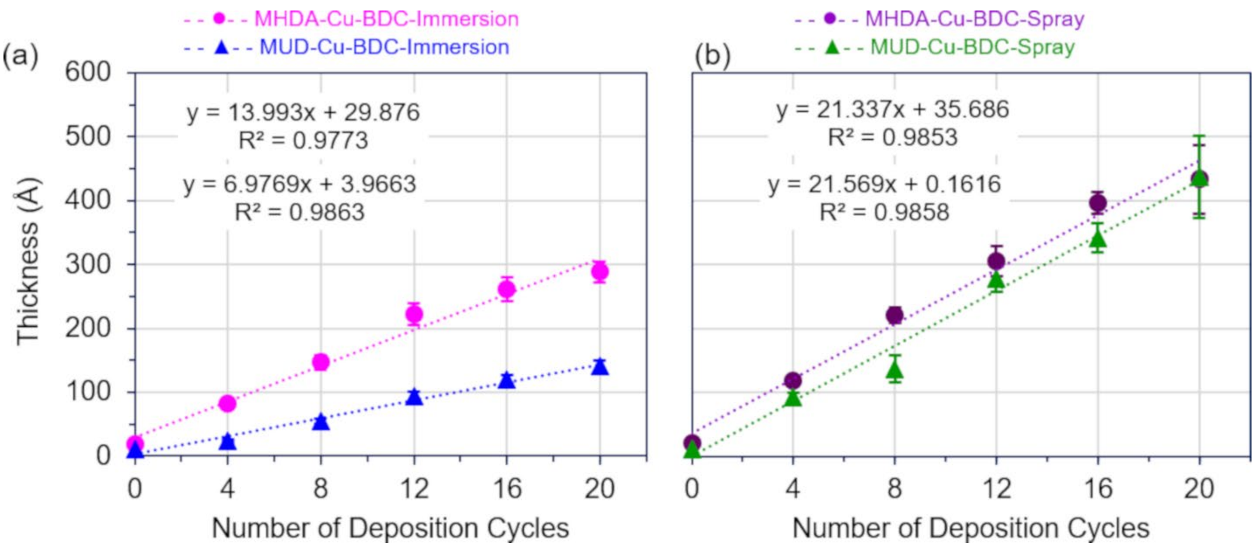

Figure S2. Ellipsometric film thicknesses, standard deviations, and corresponding linear fits are plotted as a function of deposition cycles. Films were deposited by (a) LBL immersion and (b) spray method on gold substrates functionalized with MHDA (pink or purple circles) and MUD (blue or green triangles) SAMs. This data corresponds to Figure 2b and 7b.

IR spectra.

In the image below, two representative full IR spectra from 600 to 4000 cm^{-1} are provided. Peaks around 2900 cm^{-1} correspond to the C–H stretch of the underlying self-assembled monolayer on the gold substrate.[1-2] The samples after 8 cycles of LBL immersion deposition were selected because there was significant amount of film to clearly observe peaks for the MOF while still being thin enough to collect signal associated with the underlying SAM.

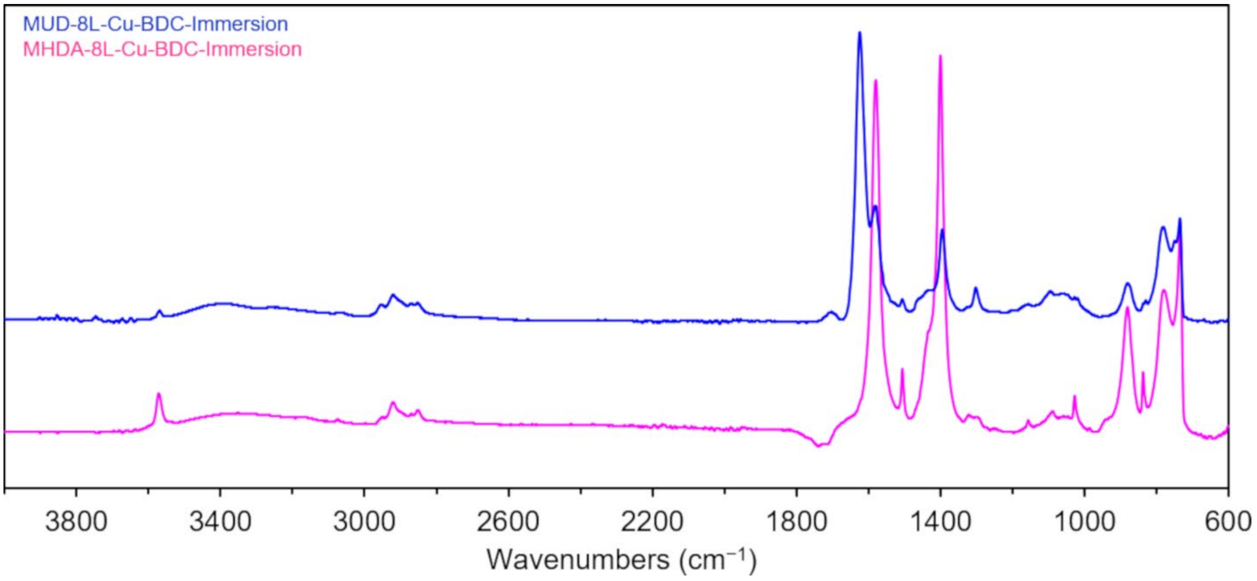

Figure S3. Representative infrared spectra for Cu-BDC films deposited by 8 LBL immersion deposition cycles on MHDA (pink, bottom) and MUD (blue, top) SAMs.

Morphology, thickness, and crystallinity of the films deposited using LBL immersion method on MHDA and MUD SAMs assembled for 24 Hr and 1Hr, respectively. Provided for comparison to 1 Hr MHDA and 24 Hr MUD SAMs in manuscript.

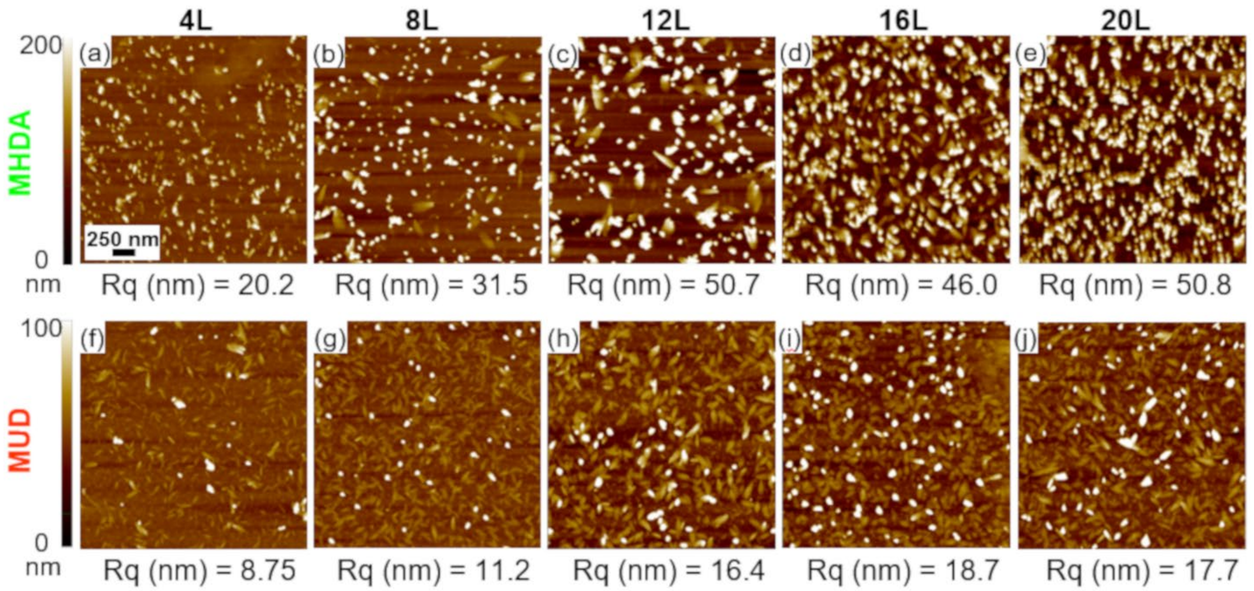

Figure S4. Representative atomic force microscopy images (2.5 μm x 2.5 μm) of Cu-BDC films deposited by LBL immersion on 24 Hr MHDA (a-e) and 1 Hr MUD (f-j) SAMs. Above each image, the number of LBL deposition cycles (L) completed prior to analysis is given. Below each image, the corresponding surface roughness value (Rq) is provided and is specific to the AFM image. Scale bar of 250 nm (a) is for all images. In each row all the images are set to the same *z*-scale shown to left of images (a) and (f). For comparison, see Figure 1 in manuscript.

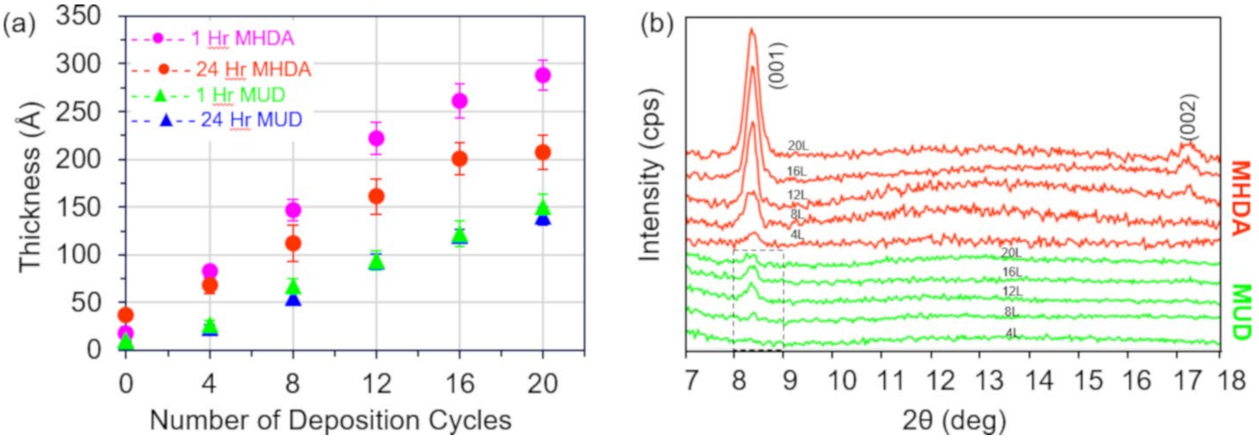

Figure S5. Films were deposited by LBL immersion method on gold substrates functionalized with MHDA and MUD SAMs. (a) Average thickness and corresponding standard deviation values of films deposited on 1 Hr MHDA (pink circles), 24 Hr MHDA (orange circles), 1 Hr MUD (green triangles), and 24 Hr MUD (blue triangles) are plotted as a function of deposition cycles. (b) XRD patterns of Cu-BDC films deposited on 24 Hr MHDA (orange, top set) and 1 Hr MUD (green, bottom set) SAMs. For comparison, see Figure 2 and 3 in manuscript.

Stability experiments.

Four tests were performed on Cu-BDC films deposited by 20 LBL immersion deposition cycles on gold substrates functionalized with MHDA or MUD SAMs to study the stability of the film structure, morphology, and composition under different environmental conditions. A tape test was performed to determine adhesive properties of the film. A transparent piece of Scotch tape was placed over the film, pressed gently with a finger, and peeled off immediately. Water stability was tested by immersing films in de-ionized water for 1 min and 1 hr. Thermal stability was examined by heating at 110°C under vacuum for 1 hr. Samples were analyzed using XRD (Figure S6), ellipsometry (Table S1), and AFM (Figure S7), and before and after each experiment. Cu-BDC films deposited on MHDA are stable against water and heat; however, they are not well adherent to the surface. On the other hand, films deposited on MUD are well adherent as well as stable in water and under vacuum at 110°C.

Tape test was performed for Cu-BDC films deposited by 16 and 20 LBL spray deposition cycles on MHDA and MUD functionalized gold substrates to study film adhesive properties. Film thickness was determined before and after the experiment and results are provided in Table S2. No differences are observed in the before and after thicknesses confirming strong adhesive properties of the films deposited by LBL spray method. For the Cu-BDC deposited on MHDA SAM, results differ for tape test depending on LBL immersion or spray method. This is consistent with the different morphologies observed by AFM (Figure 1 and 6) for the samples by different deposition methods. This suggests that the larger island-type particles by spray are more adhesive to the substrate than standing up crystallites obtained by immersion method on MHDA SAMs. This further demonstrates how the crystal orientation affects the adhesion stability of the films.

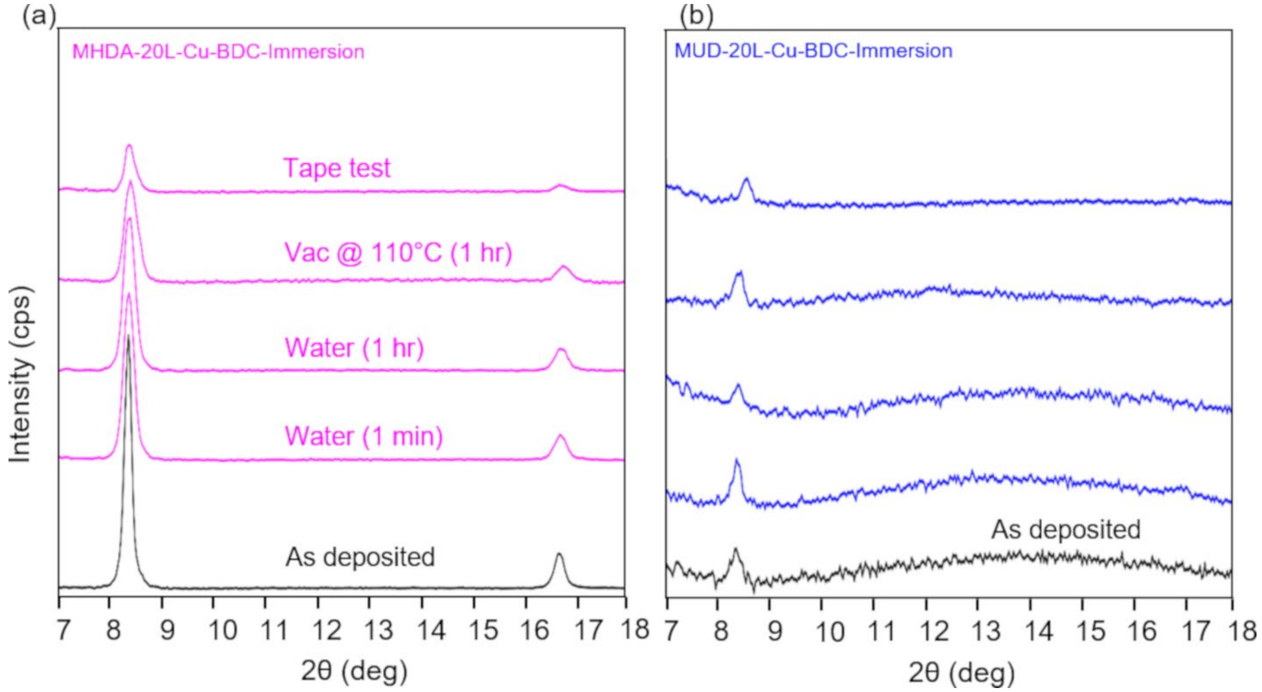

Figure S6. XRD patterns of Cu-BDC films deposited by 20 LBL immersion deposition cycles on MHDA and MUD SAMs. Patterns collected for as-deposited samples are shown in black. Patterns after stability experiments are displayed in pink for samples on MHDA (a) and blue for samples on MUD (b). These XRD patterns are collected for samples with AFM images and thickness values given in Figure S7 and Table S1, respectively.

Table S1. Ellipsometric thickness and associated standard deviation values for Cu-BDC films deposited by 20 LBL immersion deposition cycles (L) before and after stability experiments. These thicknesses are for samples with AFM images shown in Figure S7.

	**20 L film deposited on MHDA**		**20 L film deposited on MUD**	
**Condition**	**Thickness Before (Å)**	**Thickness After (Å)**	**Thickness Before (Å)**	**Thickness After (Å)**
Water (1 min)	277±47	256±36	160.±33	194±23
Water (1 hr)	300.±37	297±43	154±14	138±30.
Tape test	290.±59	96±72	172±25	174±16
Vac @ 110°C (1 hr)	269±25	259±43	178±21	156±17

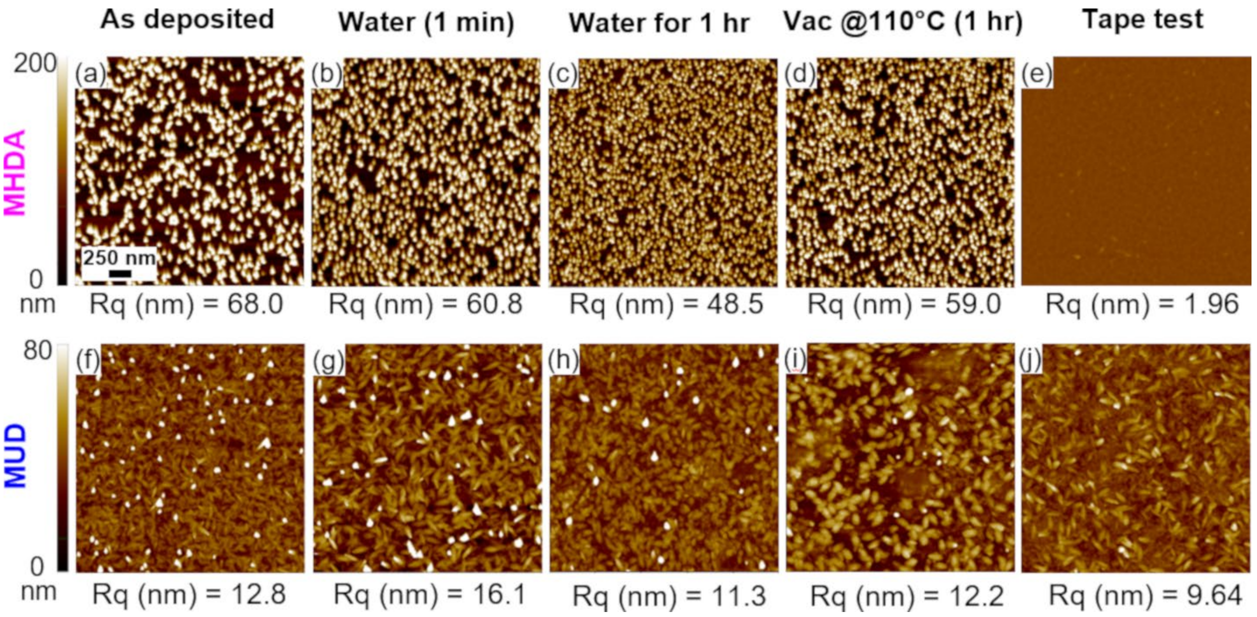

Figure S7. Representative atomic force microscopy images (2.5 µm x 2.5 µm) of Cu-BDC films deposited by 20 LBL immersion deposition cycles on MHDA (a–e) and MUD (f–j) SAMs collected before (a,f) and after (b–e, g–j) stability experiments. Below each image, the corresponding surface roughness values (Rq) are provided. Above each column of images is a brief description of the experiment. Scale bar of 250 nm (a) is for all the images. In each row all the images are set to the same z-scale shown to left of images (a) and (f).

LBL spray deposition.

"CENTRAL PNEUMATIC" air brush compressor kits were used to spray Cu-BDC on MHDA and MUD functionalized gold substrates. The air brush nozzle size was 0.35 mm. The nozzle-sample distance was maintained at 6.5 inches. Working air pressure was 60 psi. One deposition cycle includes 20 s metal deposition, ethanol rinse, dry under a nitrogen flow, 20 s organic deposition, ethanol rinse, and dry under a nitrogen flow. Ethanol rinsing step was carried out using a laboratory squeeze bottle. Around 1mL of ethanol was used to rinse over approximately 6 seconds. Gold substrate sample size was 1x1 cm minimum.

XRD patterns of LBL spray deposited samples.

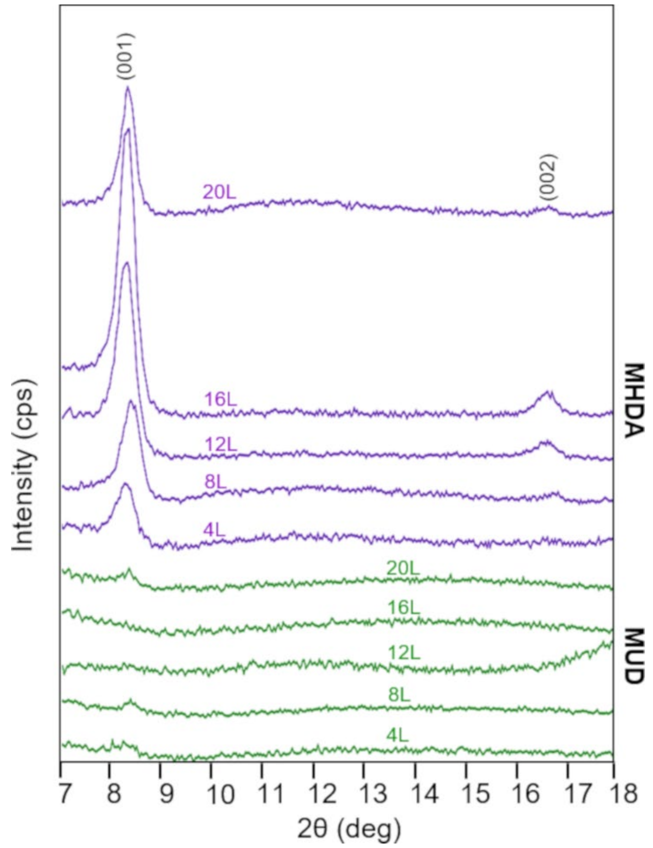

Figure S8. XRD patterns of Cu-BDC films deposited by LBL spray method on MHDA (purple, top set) and MUD (green, bottom set) SAMs. The number of LBL deposition cycles (L) is represented above the corresponding pattern.

Table S2. Ellipsometric thickness and associated standard deviation values for Cu-BDC films deposited by 16 and 20 LBL spray deposition cycles (L) before and after tape test.

	**Film deposited on MHDA**		**Film deposited on MUD**	
**Tape test**	**Thickness Before** (Å)	**Thickness After** (Å)	**Thickness Before** (Å)	**Thickness After** (Å)
16L Cu-BDC	428±31	431±37	356±42	388±34
20L Cu-BDC	518±142	507±166	483±194	426±189

Scanning electron microscopy (SEM).

Initial investigation of the samples by SEM was conducted using a JEOL JSM IT-200LA microscope. An accelerating voltage of 15 keV, probe current ranging from 30–40 μA, and working distance ranging from 7.5–11 mm were used to acquire images.

As shown in Figure S9 (a,f), standing-up crystallites are observed by both AFM and SEM for films deposited using immersion method on MHDA. For films deposited on MUD by immersion, some standing-up features consistent with AFM are observed, but lying-down rod-shaped crystallites are not clearly resolved by SEM (Figure S9b,g). Both AFM and SEM images of the LBL spray deposition samples (Figure S9c,d and h,i) show less distinct morphological structure than observed for the LBL immersion method. SEM images for the spray samples show similar features sizes to those observed by AFM. These images were obtained using a conventional tungsten electron-beam source SEM. Future research using a field emission SEM would be required to further resolve crystal structure and size.

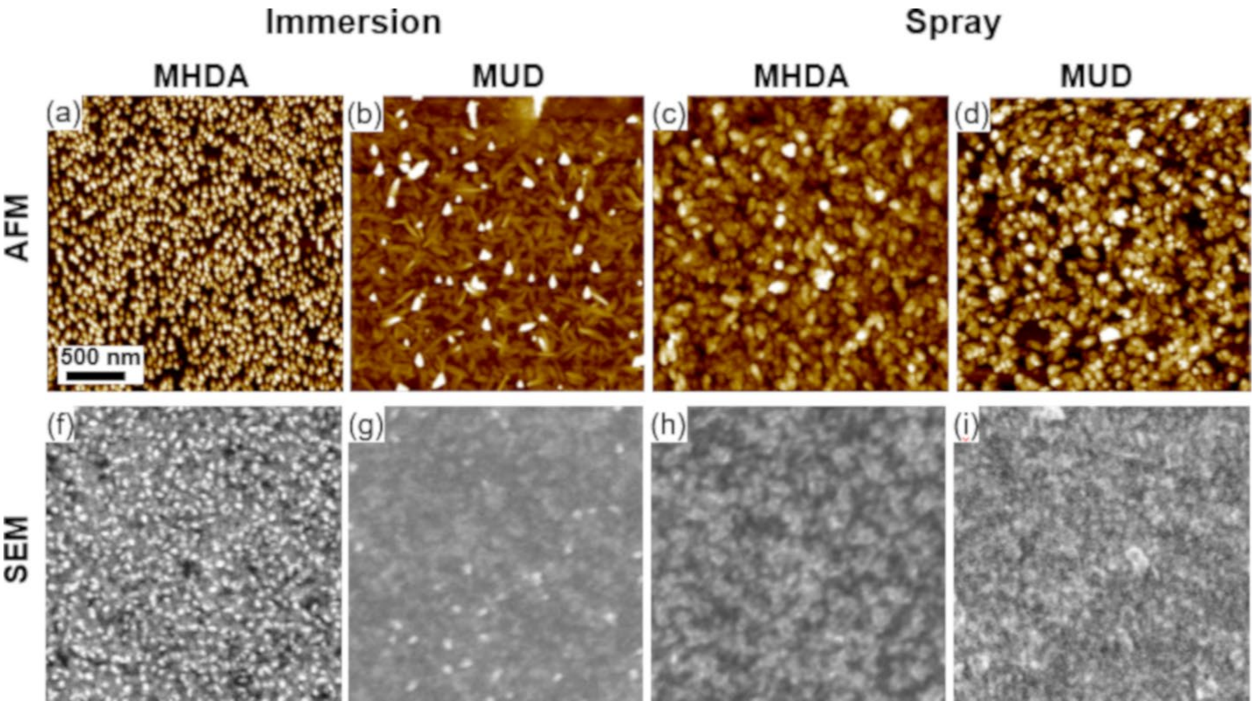

Figure S9. Top: Atomic force microscopy images (2.5 µm x 2.5 µm) of Cu-BDC films deposited by 20 deposition cycles of LBL immersion (a, b) and LBL spray (c, d) methods on MHDA (a, c) and MUD (b, d) SAMs. These AFM images are also found in manuscript Figure 1 and 6. Bottom: SEM images (2.5 µm x 2.5 µm) of Cu-BDC films deposited by 20 deposition cycles of LBL immersion (f, g) and LBL spray (h, i) methods on MHDA (f, g) and MUD (h, i) SAMs.

Reference:

1. Daniel, T. A.; Uppili, S.; McCarty, G.; Allara, D. L., Effects of Molecular Structure and Interfacial Ligation on the Precision of Cu-Bound α,ω-Mercaptoalkanoic Acid "Molecular Ruler" Stacks. *Langmuir* **2007,** *23* (2), 638-648.

2. Bowser, B. H.; Brower, L. J.; Ohnsorg, M. L.; Gentry, L. K.; Beaudoin, C. K.; Anderson, M. E. Comparison of Surface-Bound and Free-Standing Variations of HKUST-1 MOFs: Effect of Activation and Ammonia Exposure on Morphology, Crystallinity, and Composition *Nanomaterials* **2018**, *8*, 650-667.
